# Relationship between a pressure redistributing foam mattress and pressure injuries: An observational prospective cohort study

**DOI:** 10.1371/journal.pone.0241276

**Published:** 2020-11-09

**Authors:** Dorothy Li Bai, Tsai-Wen Liu, Hsiu-Ling Chou, Yeh-Liang Hsu

**Affiliations:** 1 Gerontechnology Research Center, Yuan Ze University, Taoyuan, Taiwan; 2 Department of Nursing, Far Eastern Memorial Hospital, New Taipei City, Taiwan; 3 Department of Nursing, Oriental Institute of Technology, New Taipei City, Taiwan; 4 School of Nursing, National Yang-Ming University, Taipei, Taiwan; 5 Mechanical Engineering Department, Yuan Ze University, Taoyuan, Taiwan; University of Ottawa, CANADA

## Abstract

**Background and purpose:**

Pressure injuries remain a significant health care issue in various settings. The purpose of this study was to examine the relationship between a pressure redistributing foam mattress (PRFM) and the development of pressure injuries.

**Methods:**

This study employed an observational prospective cohort study design. We enrolled 254 participants from the intensive care unit who were at risk of developing pressure injuries. Participants were exposed to either a nonpressure redistributing foam mattress (NPRFM), which was the standard mattress used at the study site, or a PRFM made of viscoelastic, temperature-sensitive, polyurethane memory foam. The patients’ assignment to either a PRFM or NPRFM was performed upon their admission, before the study eligibility screening. The relationship between the PRFM and the development of pressure injuries was studied using a logistic regression model.

**Results:**

The overall incidence of pressure injuries was 5.9% (15/254) in our study, with 1.6% (2/127) for participants who used a PRFM and 10.2% (13/127) for those using a NPRFM. After adjusting for potential confounding variables, use of a PRFM was associated with an 88% reduced risk of pressure injury development (OR = 0.12, 95% CI: 0.03, 0.56, P = 0.007). The use of a PRFM also contributed to a postponed occurrence of pressure injuries by 4.2 days on average in comparison with that of a NPRFM (*P* = 0.041).

**Conclusions:**

A PRFM is associated with a significantly reduced incidence and postponed occurrence of pressure injuries. It is recommended to use a PRFM for patients at risk of developing pressure injuries.

## Introduction

A pressure injury is defined as an area of localized damage to the skin and underlying tissue mainly caused by pressure, or pressure in combination with shear, which usually develops on bony prominences, such as the sacrum, coccyx bone and heel [1]. Its prevalence varies widely according to the stage of injury, population characteristics, and health care settings, with the overall prevalence of inpatient pressure injuries ranging from 2.9% to 23% [2–6]. Pressure injuries are associated with several significant physical, psychological and social difficulties for individuals [7], a negatively affected quality of life [8, 9], and increased mortality [10]. In addition, they also create a significant burden on the health care system and society. Evidence suggests that pressure injuries extended the length of stay by 2 to 50 days [11, 12]. The annual cost to treat pressure injuries is approximately 1.4–2.1 billion pounds in the UK and 2.2–3.6 billion dollars in the US [13, 14]. Dealey et al. found that the cost was influenced by the severity of the wound, costing 1,214 pounds per patient for Stage I and 14,108 pounds per patient for Stage II pressure injuries [15].

A wide range of factors have been indicated to be associated with pressure injuries, such as age, immobility, malnutrition, lower blood hemoglobin, lower serum albumin, higher white blood cell count, and most importantly, surfaces (e.g., beds, seating) without appropriate pressure relief [16, 17]. Particularly, patients in the intensive care unit (ICU) are often sedated and confined to bed and are therefore at a higher risk of developing pressure injuries [18, 19]. The incidence rate of pressure injuries in the ICU has been reported in a broad range, from 2.6% to 56% [4, 5, 14, 20]. The likelihood of developing pressure injuries is 3.8 times higher in intensive care settings than that in nonintensive care settings [21].

Although considerable effort has been made in education, training, and prevention equipment, pressure injuries remain a significant health care issue in various settings [22]. Regular manual repositioning (e.g., two-hour turning) is an effective strategy for preventing pressure injuries by removing or redistributing pressure from a particular part of the body [19]. Pressure redistribution prevents or treats pressure injuries by decreasing the magnitude and/or duration of the interfacial pressure [1]. Apart from manual repositioning, a pressure-relieving support surface is an alternative way to redistribute pressure by reducing the shear or friction between the user and the surface [2, 23, 24]. Although air-filled and water-filled support surfaces are used in specific situations to prevent pressure injuries, they are neither cost-effective nor practical [2, 25]. Alternatively, foam mattresses or overlays, especially those with pressure redistributing characteristics, have become an increasingly popular method of attempting to reduce the development of pressure injuries without compromising patients’ comfort [2, 25]. However, the findings on the association between foam supports and the occurrence of pressure injuries are inconsistent, with some showing a decrease in the incidence of pressure injuries [26–29], some concluding no significant effect [30–32], and some presenting a slight increase in the development of pressure injuries [33] depending on various characteristics of the support surfaces, study design, settings, etc.

The purpose of this study was to examine the relationship between a pressure redistributing foam mattress (PRFM) and the development of pressure injuries in an intensive care unit. The primary objectives were to compare the incidence, development time, and severity of pressure injuries between ICU patients who used a PRFM and those who used a nonpressure redistributing foam mattress (NPRFM).

## Methods

### Study design and participants

This study employed an observational prospective cohort study design. A sample of 254 participants from the ICU at a medical center in Taiwan were recruited from November 2017 to September 2018 and followed up until they were discharged from the ICU. The inclusion criterion was the risk of pressure injury development as identified using the Braden pressure injury risk assessment scale (≤16) [34]. The Braden scale was used to assess the risk of pressure by measuring sensory, moisture, activity, mobility, nutrition and friction/shear; it is one of the most frequently used tools for pressure injury assessment in clinical practice [34]. The cutoff points for the risk of developing pressure injuries are as follows: low risk (total score: 15–18), moderate risk (total score: 13–14), high risk (total score: 10–12), and very high risk (total score: ≤ 9) [35]. The exclusion criteria were pressure injuries at the time of recruitment, medical conditions that would preclude the use of repositioning, and the use of an air cushion bed. Study procedures were reviewed and approved by the Research Ethics Review Committee of Far Eastern Memorial Hospital with approval number FEMH-105119-F. Written informed consent was obtained from each participant or legal representative.

### Study procedures and instruments

There were 30 beds in the ICU; one-third of them had a PRFM (n = 10) and the others (n = 20) had a NPRFM, which were the standard mattresses used at the study site. To minimize the clinical inconvenience and possible research bias, patients’ assignment to either a PRFM or NPRFM was performed upon their admission to the ICU, before the study eligibility screening. Administrative nurses conducted the mattress assignment according to standard operating procedures, without notification or training for this study. The bed occupancy rate was approximately 92% in the ward, and newly admitted patients were randomly assigned to an empty bed, which helped to ensure a quasi-randomization of the exposure to the mattresses. In addition, the assignment of nursing staff was according to patients’ discharge and admission situations instead of beds, so it is less likely that a specific nursing staff member was consistently assigned to certain beds with a PRFM or NPRFM. Participants received standard nursing care but used different mattresses. The PRFM was a commercially available mattress from SEDA Chemical Co., Ltd. (Product Model: IMAGER-37^®^AM-200) which utilizes a viscoelastic, polyurethane foam material. The NPRFM was a polyurethane foam mattress from Chang Gung Medical Technology Co., Ltd. (Product Model: GM01) and was not designed to redistribute pressure.

The major differences between the PRFM and NPRFM include thickness, density, firmness (or stiffness), and elasticity, which have been shown to be factors that affect the pressure-distributing properties of foam mattresses [25, 36]. The thickness and density are related to the support characteristics of a foam mattress, with higher thickness and density providing better support of the body [25]. The PRFM was 12 cm in thickness with a density of 90.0 kg/m^3^ for the top layer and 55.3 kg/m^3^ for the bottom layer. The NPRFM was 10 cm in thickness with a density of 30.2 kg/m^3^ for the top layer and 40.1 kg/m^3^ for the bottom layer. The firmness of a foam mattress is usually measured by the indentation force deflection (IFD), with a higher score indicating a greater firmness [36]. The sag factor (modulus) is the ratio of the 65% IFD to the 25% IFD values and provides an indication of cushioning quality. The higher the modulus is, the better the cushioning quality of the mattress [36]. The IFD of the PRFM was 10.5 kg/314 m^3^ at 25% and 36.9 kg/314 m^3^ at 65%, resulting in a modulus of 3.5. The IFD of the NPRFM was 17.7 kg/314 m^3^ at 25% and 35.2 kg/314 m^3^ at 65%, resulting in a modulus of 2.0. Impact resilience is a measure of the elasticity or springiness of foam by dropping a standard steel ball onto the foam from a given height and is expressed as the percentage of the distance the ball rebounds [36]. Low resilience represents a low spring-back property and may be associated with reduced mechanical force from a tangential load [37]. The impact resilience of the PRFM and NPRFM was 0.5% and 23%, respectively. In addition, the PRFM is also temperature sensitive, especially at approximately 37 degrees Celsius, when it slowly softens to shape to the figure of the body. The mechanical data showed that the PRFM has better support properties, a higher cushioning quality, and better elasticity than those of the NPRFM. These physical characteristics of the PRFM help to achieve a maximum contact area between the body and the mattress surface and reduce the potential mechanical force by the mattress to effectively reduce the interface pressure [38].

### Variables

The primary outcome variable was the development of pressure injuries. In addition to the type of mattress, several confounding variables were included, such as age (in years), sex, risk of pressure injury development (Braden score), bedridden prior to admittance into the ICU, length of stay in the ICU, and the number of comorbidities. To assess the severity of pressure injuries, we used the guidelines of the pressure injury classification system by the National Pressure Injury Advisory Panel, European Pressure Injury Advisory Panel, and Pan Pacific Pressure Injury Alliance [1]. According to the severity of the injury, pressure injuries are grouped as Stage I (non-blanchable erythema), Stage II (partial-thickness skin loss), Stage III (full-thickness skin loss), Stage IV (full-thickness tissue loss), unstageable (depth unknown), and suspected deep tissue injury (depth unknown) [1].

### Data analysis

A descriptive analysis was used to examine the characteristics of participants, using mean, frequency, and percentage depending on variables, as well as the occurrence time, location, stage, and range of the injury. Chi-squared tests and t-tests were conducted to compare the characteristics of participants using different mattresses. Chi-squared tests were applied to compare whether there were significant differences in the occurrence of pressure injuries between participants using PRFM and those using NPRFM. In addition, unadjusted and adjusted logistic regression models were used to investigate the relationship between a PRFM and the development of pressure injuries. The Hosmer–Lemeshow goodness-of-fit test was conducted to assess the adequacy of the logistic models, and a variance inflation factor was used to assess multicollinearity [39, 40]. All data analyses were performed using the statistical software package Stata Statistical Software: Release 16 (College Station, TX: StataCorp LLC) [41]. A nominal significance level of 0.05 and power of 80% were used throughout the analysis.

When calculating the sample size for a multiple logistic regression model, the rule of ten events per variable is widely used [42], and recently, the rule of 20 or 50 events per variable has been increasingly suggested [43]. However, it can be difficult when the event is rare, such as the occurrence of pressure injuries in our study. The sample size calculation for this type of study is based on the upper limit of risk, which is computed as 3, divided by the sample size [44]. Given that the incidence of pressure injuries at the study site was between 1.5% and 12.3% (unpublished internal report), the sample size was estimated to include 200 participants.

## Results

**Fig 1:**
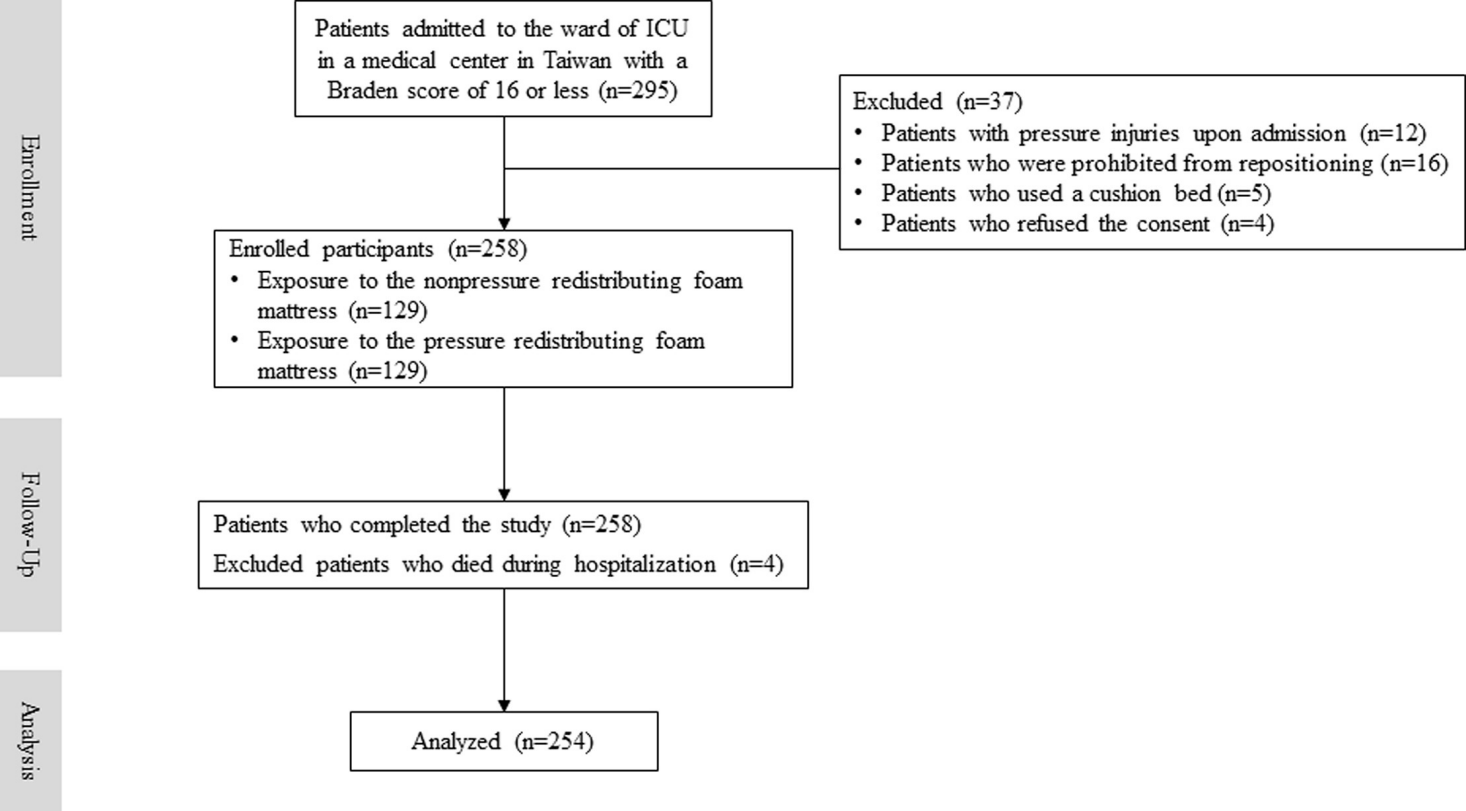
Flow diagram of participants.

We recruited, followed-up, and analyzed the data of 254 participants from the ICU for the investigation of pressure injuries in this study (Fig 1). The characteristics of participants were compared using different mattresses, and no significant variations were found (Table 1). Among all participants, half (n = 127) used a NPRFM and the other half (n = 127) used a PRFM. The total incidence of pressure injuries in our study was 5.9% (15/254). Furthermore, we observed that the incidence of pressure injuries among participants using a PRFM was 1.6%, which was lower than that of 10.2% among those using a NPRFM, with statistical significance (*P* = 0.003). The average age of all participants was 64 years, and approximately 61% were male. The average stay in the ICU for all participants was 8.9 days, with no significant differences between participants who used a PRFM and a NPRFM.

**Table 1:** Participant characteristics.

	Total n (%)	NPRFM^a^ n (%)	PRFM^b^ n (%)	*P* value
Pressure injury				.003
No	239 (94.1)	114 (89.8)	125 (98.4)	
Yes	15 (5.9)	13 (10.2)	2 (1.6)	
Age (mean)	64.05	64.4	63.7	.745
Sex				.302
Male	156 (61.4)	74 (58.3)	82 (64.6)	
Female	98 (38.6)	53 (41.7)	45 (35.4)	
Braden score				.715
Low risk	57 (22.4)	27 (21.3)	30 (23.6)	
Moderate risk	84 (33.1)	39 (30.7)	45 (35.4)	
High risk	73 (28.7)	40 (31.5)	33 (26.0)	
Very high risk	40 (15.8)	21 (16.5)	19 (15.0)	
Bedridden before admission				.472
No	246 (96.8)	124 (97.6)	122 (96.1)	
Yes	8 (3.2)	3 (2.4)	5 (3.9)	
Length of stay in the ICU (average days)	8.90	8.34	9.46	.122
Number of comorbidities				.586
0	26 (10.2)	14 (11.0)	12 (9.5)	
1	71 (28.0)	33 (26.0)	38 (29.9)	
2	54 (21.3)	31 (24.4)	23 (18.1)	
≥ 3	103 (40.5)	49 (38.6)	54 (42.5)	

^a^NPRFM: Nonpressure redistributing foam mattress

^b^PRFM: Pressure redistributing foam mattress

The characteristics of pressure injuries are described in Table 2. In total, 15 participants developed pressure injuries, of whom three had more than one injury at the same time. Of all participants who developed pressure injuries, nine (60%) had an injury in the coccyx, while the other six developed pressure injuries in different locations, including one each (6.7%) at the buttock, ankle, greater trochanter, shoulder, back, and occiput. The injuries ranged in size from 0.5*0.2 cm to 10*10 cm, depending on the location and severity.

**Table 2:** Characteristics of pressure injuries.

Mattress	Pressure injury 1				Pressure injury 2 (if any)			
	Occurrence time (days)	Location	Stage	Range (cm)	Occurrence time (days)	Location	Stage	Range (cm)
NPRFM^a^	6	Buttock	I	2*2				
NPRFM^a^	7	Ankle	II	0.5*0.2				
NPRFM^a^	5	Coccyx	I	5*5				
NPRFM^a^	1	Greater trochanter	I	6*3				
NPRFM^a^	11	Coccyx	I	5*5	11	Coccyx	II	2*2
NPRFM^a^	2	Coccyx	I	5*5				
NPRFM^a^	8	Shoulder	II	2*1				
NPRFM^a^	3	Back	II	6*4				
NPRFM^a^	1	Coccyx	I	5*5				
NPRFM^a^	3	Coccyx	I	8*8	3	Coccyx	II	3*3
NPRFM^a^	2	Coccyx	I	8*8				
NPRFM^a^	5	Coccyx	I	10*10				
NPRFM^a^	2	Occiput	I	5*5				
PRFM^b^	8	Coccyx	I	5*5	8	Coccyx	II	2*1
PRFM^b^	9	Coccyx	II	2*2				

^a^NPRFM: Nonpressure redistributing foam mattress

^b^PRFM: Pressure redistributing foam mattress

Unadjusted and adjusted logistic regression models were employed to investigate the relationship between the mattress and the development of pressure injuries (Table 3). We found that the likelihood of developing pressure injuries was reduced by 86% (OR = 0.14, 95% CI: 0.03, 0.64, *P* = 0.011) in participants who used a PRFM when compared with those using a NPRFM. After adjusting for several confounding variables, this result remained significant and showed that the PRFM was associated with an 88% reduced risk of pressure injury development (OR = 0.12, 95% CI: 0.03, 0.56, *P* = 0.007). Additionally, a longer length of stay in the ICU was significantly associated with a higher risk of pressure injury development after adjusting for other variables (OR = 1.08, 95% CI: 1.02, 1.14, *P* = 0.010). The average length of stay in participants who did not develop pressure injuries was 8.6 days, compared to 14.3 days among participants who developed pressure injuries (*P* = 0.002). The linear regression results (data not shown in the table) showed that the development of a pressure injury prolonged the length of stay by 5.78 days (95% CI: 1.83, 9.72, *P* = 0.004). The results of the Hosmer–Lemeshow goodness-of-fit tests for the adjusted logistic model indicated that the model was a good fit for the data. Variance inflation factor values also indicated a low degree of multicollinearity.

**Table 3:** Unadjusted and adjusted Odds Ratios (OR) of development of pressure injuries according to participants’ characteristics.

	Unadjusted			Adjusted		
	OR	95% CI	*P* value	OR	95% CI	*P* value
Mattress						
NPRFM^a^	1.00	—	—	1.00	—	—
PRFM^b^	0.14	0.03–0.64	.011	0.12	0.03–0.56	.007
Age (mean)	0.99	0.96–1.03	.722	1.00	0.95–1.04	.819
Sex						
Male	1.00	—	—	1.00	—	—
Female	1.89	0.66–5.39	.233	1.81	0.56–5.84	.319
Braden score						
Low risk	1.00	—	—	1.00	—	—
Moderate risk	0.70	0.19–2.57	.590	0.70	0.17–2.96	.632
High risk	0.99	0.27–3.68	.993	0.83	0.20–3.52	.801
Very high risk	0.89	0.10–7.86	.913	0.77	0.05–11.96	.855
Length of stay in the ICU (average days)	1.07	1.02–1.12	.009	1.08	1.02–1.14	.010
Number of comorbidities						
0	1.00	—	—	1.00	—	—
1	1.31	0.25–6.77	.745	1.56	0.27–9.06	.623
2	0.23	0.02–2.62	.234	0.26	0.02–3.37	.300
≥ 3	0.61	0.11–3.35	.572	0.57	0.07–4.33	.587

^a^NPRFM: Nonpressure redistributing foam mattress

^b^PRFM: Pressure redistributing foam mattress

In addition to the occurrence of pressure injuries, we also compared the occurrence time of the first pressure injury after recruitment. The results showed that the average time to develop the first pressure injury was 4.2 days earlier among participants using a NPRFM (4.3 days) compared to those using a PRFM (8.5 days). The average time to develop a pressure injury for participants who used a PRFM was delayed compared to those who used a NPRFM, with statistical significance (*P* = 0.041). In addition, we measured and compared the severity of the pressure injuries. Among the three injuries developed by the two participants who used a PRFM, two were classified as Stage II and one as Stage I. Among the 15 injuries developed by the 13 participants who used a NPRFM, five were classified as Stage II and ten as Stage I. However, the difference in the severity of pressure injuries was statistically insignificant (*P* = 0.280).

## Discussion

The present study investigated the relationship between a PRFM and the development of pressure injuries in an intensive care unit. The total incidence of pressure injuries was 5.9% in our study. Among the participants, 1.6% of the patients using a PRFM developed pressure injuries, compared with 10.2% who used a NPRFM. Compared to the NPRFM, the PRFM was associated with an 88% reduced likelihood of developing pressure injuries. We also found that the PRFM contributed to a postponed occurrence of pressure injuries by 4.2 days on average in comparison with the NPRFM.

A pressure injury is defined as an area of localized damage to the skin and underlying tissue mainly caused by pressure, or pressure combined with shear [1]. Shear is a mechanical force created from a tangential load that causes the body to slide against the resistance between a contact surface and the skin. Friction is another mechanical force that occurs when two surfaces move across one another, creating resistance between the contact surface and the skin [1]. When shear occurs, the dermis and epidermis remain stationary, while the skeleton slides with the deep fascia. This can create deformation in the lymphatic system and in the blood vessels and lead to capillary occlusion and thrombosis [45]. Tissue tolerance for pressure and oxygen concentration also play a role in this process. It is commonly assumed that the external pressure between the skin and a supporting surface of over 32 mmHg (4.27 kPa) occludes the blood vessels and produces ischemia, which, if applied for a critical period, could result in tissue necrosis and the occurrence of a pressure injury [46]. Evidence also shows that it requires lower external pressure to cause the reduction or occlusion of skin blood flow at bony prominences compared to other regions [47]. This is one of the main reasons why pressure injuries usually develop on bony prominences, such as the sacrum, coccyx bone and heel; the well-known pressure injury predilection areas [48]. Our study consistently showed that of all pressure injuries, 60% developed in the coccyx.

Factors that increase the magnitude and duration of mechanical loading contribute to the formulation of pressure injuries [18, 49]. Unfortunately, patients from the ICU are more likely to be exposed to these factors, including but not limited to reduced activity and mobility or even being bedridden, loss of sensory perception as a result of medication such as anesthetics, sedatives, and analgesics that are commonly used in ICU patients, and maceration of skin due to sweating, incontinence, or leaking wounds [18, 50, 51]. These factors are present among patients from other departments in the hospital, while they are mostly presented at a higher level of severity or a combination of more than two factors among ICU patients. The likely worldwide prevalence range of pressure injuries in acute care settings is between 6% and 54%, and the incidence of pressure injuries is between 3% and 29% [4, 21, 52]. Coyer et al. found that patients from the ICU were 3.8 times more likely to develop pressure injuries than those from nonintensive departments [21]. In the present study, all participants recruited were from an intensive care unit, and the overall incidence of pressure injury development was 5.9%. The incidence among participants using the NPRFM was 10.2%, which is similar to previously reported results [21, 52].

Traditional two-hour turning prevents pressure injuries by shortening the constant duration of comprehensive and shearing forces. However, it mainly depends on human resources, which is generally one of the most intensive resources in hospitals [53]. A study in an intensive care unit suggested that only 36.8% of the patients who should be manually turned were actually repositioned [54]. Another major determinant of the intensity or magnitude of pressure and shearing forces is the support surface [1, 2]. A variety of materials are used for support surfaces, such as air-filled supports, water-filled supports, contoured or textured foam supports, and alternative foam mattresses or overlays [2]. As the surface materials differ significantly from each other, previous studies have not reached a definite conclusion on the effect of support surfaces on preventing pressure injury development [2, 24, 26–32]. Furthermore, these studies have a high heterogeneity in study design, sample size, settings, and comparative standard hospital mattresses, which may also partially explain the heterogeneity in results.

A network meta-analysis study found that powered active air surfaces offer a reduced incidence of pressure injuries but provide compromised comfort compared with foam mattresses [24]. In general, foam mattresses provide a higher level of comfort in comparison with other construction materials and are widely used at home or in hospital settings [2]. Even among foam mattresses, there is a significant difference in their ability to reduce pressure depending upon the properties of the foam used [2]. Compared to other types of foam, viscoelastic foam, also known as memory foam, has unique properties in firmness (or stiffness), density, thickness, and elasticity [25, 36]. These physical characteristics help to achieve a maximum contact area between the body and the surface to effectively reduce the interfacial pressure [33]. In the present study, PRFM has better support properties, a higher cushioning quality, and better elasticity than those of the NPRFM. These physical characteristics of the PRFM help to achieve a maximum contact area between the body and the mattress surface and reduce the potential mechanical force by the mattress to effectively reduce the interface pressure. A mattress with the same material was investigated for its pressure-relieving effects, and the results suggested that the average body pressure was 17.2% lower compared with another standard nursing institutional mattress [38]. Similarly, Defloor measured the interfacial pressure on 62 healthy volunteers lying in 10 different positions and suggested that the use of a viscoelastic polyurethane foam mattress contributed to reducing the pressure by 20–30% compared to a standard institutional mattress (12 cm-thick cold foam) [55]. Furthermore, the interfacial pressure is distributed more evenly with a high-density foam mattress [56]. McInnes et al. conducted a pooled analysis of five studies that compared foam alternatives with a standard hospital foam mattress using a random-effects model (I^2^ = 77%), which showed a 60% reduction in the incidence of pressure injuries (95% CI: 0.21, 0.74) [2, 28–30, 32].

In this study, we also found a significant association between the length of stay and the occurrence of pressure injuries. This was consistent with the existing evidence that the length of stay in the ICU has been identified as a risk factor [57, 58]. Despite the significant results, our study and most other studies showed that the odds ratio was very close to 1 [57–59]. However, the relationship between the length of stay and the development of pressure injuries is reversible. It can also be concluded that the occurrence of a pressure injury leads to an extended length of stay. A prospective cohort study involving 4,500 participants showed that a pressure injury prolonged the length of stay by a median of 4.31 days [11]. Another study with 3,198 aged patients showed an extended length of stay of 2.6 days in patients who developed pressure injuries compared to those without pressure injuries [12]. Similarly, we observed an extended length of stay by 5.78 days in participants who developed a pressure injury when compared to those without a pressure injury.

This was a prospective cohort study with 254 participants recruited in an intensive care unit at a medical center in Taiwan. However, this study has some limitations. First, this was not a population-based study. There are several differences in the characteristics and conditions between patients in the ICU and those in other departments in the hospital [18, 50]. In addition, family members of patients with more severe conditions might not have allowed their participation in the study, and we do not have data on that part of the population. Therefore, the study findings are limited in their generalizability to the entire inpatient population. Another limitation refers to the measurement of pressure injury stages. It has been suggested to only include pressure injuries that are Stage II or above due to the low clinical severity and assessment reliability of Stage I [60]. Given that Stage I, by definition, is still considered as one of the pressure injury stages and the prevention of this injury stage remains one of the clinical performance evaluations [1], we analyzed the outcomes of any stage of pressure injury. To improve the reliability of the injury assessment, a research nurse with substantial experience in pressure injury evaluation conducted the assessment for all participants in the study. The results, however, should still be interpreted with caution.

Furthermore, we did not collect the data of the APACHE II score as a confounding variable for the relationship between the PRFM and the development of pressure injuries. However, the influence of the APACHE II score on the development of pressure injuries is inconsistent based on existing evidence [61, 62]. We included the Braden score in the analysis, which is more widely and consistently used as a significant predictor of pressure injuries [34]. The health care providers in the ICU were not blinded to the exposure of the mattresses, and this may create biases for possible confounders. For example, it was challenging to uniformly provide manual repositioning to all participants using different mattresses. It is possible that care providers would reposition patients manually more frequently using a NPRFM if they perceived the PRFM provided greater protection from pressure injuries to meet clinical performance requirements [2, 59]. Despite this, our study still showed that the PRFM was significantly associated with a reduced incidence of pressure injuries. Future studies are suggested to measure the actual practice of manual repositioning as a confounder using advanced technologies, such as motion-sensing materials, to better investigate the independent effect of the supporting surface on pressure injury development.

## Conclusions

A pressure redistributing foam mattress was associated with a significantly reduced incidence of pressure injuries and postponed the occurrence of pressure injuries without compromising patients’ comfort. Thus, in both clinical practice and research, it is recommended to use a pressure redistributing foam mattress for patients at risk of developing pressure injuries, when possible. Future studies should investigate and verify the effect of a foam mattress with similar characteristics on reducing the incidence of pressure injuries in other populations, such as general inpatients and institutional residents. In addition, the occurrence time of pressure injuries from admission is also suggested to be one of the outcome variables describing the effect, given the limited evidence. Finally, it would be interesting to study whether the mattress can be integrated with sensing technologies that can collect real-time data on the interfacial pressure between patients and the supporting surface or on the quantity/magnitude of patients’ activities in bed to more precisely investigate the effect of mattresses or other overlays on pressure injury development.

## Supporting information

S1 File STROBE statement–checklist of items that should be included in reports of cohort studies. (DOC)

S2 File Raw data underlying the findings. (XLS)

## References

[1] European Pressure Ulcer Advisory Panel, National Pressure Injury Advisory Panel, Pan Pacific Pressure Injury Alliance. Prevention and Treatment of Pressure Ulcers/Injuries: Clinical Practice Guideline. The International Guideline. Emily Haesler (Ed.). Westford, MA, USA: EPUAP-NPIAP-PPPIA: 2019.

[2] McInnes E, Jammali‐Blasi A, Bell‐Syer SE, Dumville JC, Middleton V, Cullum N. Support surfaces for pressure ulcer prevention. Cochrane Database of Systematic Reviews. 2015;(9). 10.1002/14651858.CD001735.pub5 26333288 PMC7075275

[3] Fu Shaw L, Chang P-C, Lee J-F, Kung H-Y, Tung T-H. Incidence and predicted risk factors of pressure ulcers in surgical patients: experience at a medical center in Taipei, Taiwan. BioMed research international. 2014. 10.1155/2014/416896 PMC4099038 25057484

[4] Tubaishat A, Papanikolaou P, Anthony D, Habiballah L. Pressure ulcers prevalence in the acute care setting: a systematic review, 2000–2015. Clinical nursing research. 2018;27(6):643–59. 10.1177/1054773817705541 28447852

[5] VanGilder C, Lachenbruch C, Algrim-Boyle C, Meyer S. The International Pressure Ulcer Prevalence™ Survey: 2006–2015. Journal of Wound, Ostomy and Continence Nursing. 2017;44(1):20–8. 10.1097/WON.0000000000000292 27977509

[6] Nakashima S, Yamanashi H, Komiya S, Tanaka K, Maeda T. Prevalence of pressure injuries in Japanese older people: A population-based cross-sectional study. PloS one. 2018;13(6). 10.1371/journal.pone.0198073 PMC5991732 29879151

[7] Hopkins A, Dealey C, Bale S, Defloor T, Worboys F. Patient stories of living with a pressure ulcer. Journal of advanced nursing. 2006;56(4):345–53. 10.1111/j.1365-2648.2006.04007.x 17042814

[8] Essex HN, Clark M, Sims J, Warriner A, Cullum N. Health‐related quality of life in hospital inpatients with pressure ulceration: assessment using generic health‐related quality of life measures. Wound repair and regeneration. 2009;17(6):797–805. 10.1111/j.1524-475X.2009.00544.x 19903301

[9] Gorecki C, Brown JM, Nelson EA, Briggs M, Schoonhoven L, Dealey C, et al Impact of pressure ulcers on quality of life in older patients: a systematic review. Journal of the American Geriatrics Society. 2009;57(7):1175–83. 10.1111/j.1532-5415.2009.02307.x 19486198

[10] Landi F, Onder G, Russo A, Bernabei R. Pressure ulcer and mortality in frail elderly people living in community. Archives of gerontology and Geriatrics. 2007;44:217–23. 10.1016/j.archger.2007.01.030 17317456

[11] Graves N, Birrell F, Whitby M. Effect of pressure ulcers on length of hospital stay. Infection Control & Hospital Epidemiology. 2005;26(3):293–7. 10.1086/502542 15796283

[12] Theisen S, Drabik A, Stock S. Pressure ulcers in older hospitalised patients and its impact on length of stay: a retrospective observational study. Journal of clinical nursing. 2012;21(3‐4):380–7. 10.1111/j.1365-2702.2011.03915.x 22150944

[13] Phillips L, Buttery J. Exploring pressure ulcer prevalence and preventative care. Nursing times. 2009;105(16):34–6. 19480167

[14] Catherine VanGilder M, Amlung S, Harrison P, Meyer S. Results of the 2008–2009 International Pressure Ulcer Prevalence™ Survey and a 3-year, acute care, unit-specific analysis. Ostomy Wound Manag. 2009;55:39–45. 19934462

[15] Dealey C, Posnett J, Walker A. The cost of pressure ulcers in the United Kingdom. Journal of wound care. 2012;21(6):261–6. 10.12968/jowc.2012.21.6.261 22886290

[16] Hahnel E, Lichterfeld A, Blume-Peytavi U, Kottner J. The epidemiology of skin conditions in the aged: a systematic review. Journal of tissue viability. 2017;26(1):20–8. 10.1016/j.jtv.2016.04.001 27161662

[17] Chiari P, Forni C, Guberti M, Gazineo D, Ronzoni S, D’Alessandro F. Predictive factors for pressure ulcers in an older adult population hospitalized for hip fractures: A prognostic cohort study. PloS one. 2017;12(1). 10.1371/journal.pone.0169909 PMC5222344 28068425

[18] Alderden J, Rondinelli J, Pepper G, Cummins M, Whitney J. Risk factors for pressure injuries among critical care patients: A systematic review. International journal of nursing studies. 2017;71:97–114. 10.1016/j.ijnurstu.2017.03.012 28384533 PMC5485873

[19] Moore ZE, Cowman S. Repositioning for treating pressure ulcers. Cochrane Database of Systematic Reviews. 2015;(1). 10.1002/14651858.CD006898.pub4 25561248 PMC7389249

[20] Compton F, Hoffmann F, Hortig T, Strauss M, Frey J, Zidek W, et al Pressure ulcer predictors in ICU patients: nursing skin assessment versus objective parameters. Journal of wound care. 2008;17(10):417–24. 10.12968/jowc.2008.17.10.31304 18947019

[21] Coyer F, Miles S, Gosley S, Fulbrook P, Sketcher-Baker K, Cook J-L, et al Pressure injury prevalence in intensive care versus non-intensive care patients: a state-wide comparison. Australian Critical Care. 2017;30(5):244–50. 10.1016/j.aucc.2016.12.003 28063724

[22] Smith MB, Totten A, Hickam DH, Fu R, Wasson N, Rahman B, et al Pressure ulcer treatment strategies: a systematic comparative effectiveness review. Annals of internal medicine. 2013;159(1):39–50. 10.7326/0003-4819-159-1-201307020-00007 23817703

[23] Sprigle S, Sonenblum S. Assessing evidence supporting redistribution of pressure for pressure ulcer prevention: a review. J Rehabil Res Dev. 2011;48(3):203–13. 10.1682/jrrd.2010.05.0102 21480095

[24] Shi C, Dumville JC, Cullum N. Support surfaces for pressure ulcer prevention: a network meta-analysis. PloS one. 2018;13(2). 10.1371/journal.pone.0192707 29474359 PMC5825032

[25] Krouskop TA, Noble PS, Brown J, Marburger R. Factors affecting the pressure-distributing properties of foam mattress overlays. J Rehabil Res Dev. 1986;23(3):33–9. 3772816

[26] Andersen KE, Jensen O, Kvorning SA, Bach E. Decubitus prophylaxis: a prospective trial on the efficiency of alternating-pressure air-mattresses and water-mattresses. Acta Derm Venereol (Stockholm). 1982;63:227–30. 6192636

[27] Goldstone L, Norris M, O'Reilly M, SRN JW. A clinical trial of a bead bed system for the prevention of pressure sores in elderly orthopaedic patients. Journal of advanced nursing. 1982;7(6):545–8. 10.1111/j.1365-2648.1982.tb00274.x 6759553

[28] Gray D, Campbell M. A randomised clinical trial of two types of foam mattresses. Journal of Tissue Viability. 1994;4(4):128–32.

[29] Hofman A, Geelkerken R, Wille J, Hamming J, Breslau P, Hermans J. Pressure sores and pressure-decreasing mattresses: controlled clinical trial. The Lancet. 1994;343(8897):568–71. 10.1016/s0140-6736(94)91521-0 7906329

[30] Collier M. Pressure-reducing mattresses. Journal of Wound Care. 1996;5(5):207–11. 10.12968/jowc.1996.5.5.207 8850903

[31] Gunningberg L, Lindholm C, Carlsson M, Sjödén P-O. Effect of visco-elastic foam mattresses on the development of pressure ulcers in patients with hip fractures. Journal of wound care. 2000;9(10):455–60. 10.12968/jowc.2000.9.10.26300 11933449

[32] Russell LJ, Reynolds TM, Park C, Rithalia S, Gonsalkorale M, Birch J, et al Randomized clinical trial comparing 2 support surfaces: results of the Prevention of Pressure Ulcers Study. Advances in skin & wound care. 2003;16(6):317–27. 14652518 10.1097/00129334-200311000-00015

[33] Feuchtinger J, de Bie R, Dassen T, Halfens R. A 4‐cm thermoactive viscoelastic foam pad on the operating room table to prevent pressure ulcer during cardiac surgery. Journal of clinical nursing. 2006;15(2):162–7. 10.1111/j.1365-2702.2006.01293.x 16422733

[34] Braden B, Bergstrom N. A conceptual schema for the study of the etiology of pressure sores. Rehabilitation nursing. 1987;12(1):8–16. 10.1002/j.2048-7940.1987.tb00541.x 3643620

[35] Bergstrom N. The Braden Scale for predicting pressure sore risk. Nurs res. 1987;36(4):205–10. 3299278

[36] Mills N. Polymer foams handbook: engineering and biomechanics applications and design guide: Elsevier; 2007.

[37] Soppi E, Lehtiö J, Saarinen H. An overview of polyurethane foams in higher specification foam mattresses. Ostomy/wound management. 2015;61(2):38–46. 25654780

[38] Liu Y-W, Hsu Y-L, Chang W-Y. Development of a bed-centered telehealth system based on a motion-sensing mattress. Journal of Clinical Gerontology and Geriatrics. 2015;6(1):1–8.

[39] Hair JF, Anderson RE, Babin BJ, Black WC. Multivariate data analysis: A global perspective, 7th ed. Upper Saddle River, NJ: Pearson; 2010.

[40] Hosmer DW, Hosmer T, Le Cessie S, Lemeshow S. A comparison of goodness‐of‐fit tests for the logistic regression model. Statistics in medicine. 1997;16(9):965–80. 10.1002/(sici)1097-0258(19970515)16:9&lt;965::aid-sim509&gt;3.0.co;2-o 9160492

[41] StataCorp. Stata Statistical Software: Release 16. College Station, TX: StataCorp LLC; 2019.

[42] Peduzzi P, Concato J, Kemper E, Holford TR, Feinstein AR. A simulation study of the number of events per variable in logistic regression analysis. Journal of clinical epidemiology. 1996;49(12):1373–9. 10.1016/s0895-4356(96)00236-3 8970487

[43] Vittinghoff E, McCulloch CE. Relaxing the rule of ten events per variable in logistic and Cox regression. American journal of epidemiology. 2007;165(6):710–8. 10.1093/aje/kwk052 17182981

[44] Peat J, Mellis C, Williams K, Xuan W. Calculating the sample size. Health science research, a handbook of quantitative methods Australia: Allen and Unwin. 2001:128–47.

[45] Kottner J, Black J, Call E, Gefen A, Santamaria N. Microclimate: a critical review in the context of pressure ulcer prevention. Clinical Biomechanics. 2018;59:62–70. 10.1016/j.clinbiomech.2018.09.010 30199821

[46] Bouten CV, Oomens CW, Baaijens FP, Bader DL. The etiology of pressure ulcers: skin deep or muscle bound? Archives of physical medicine and rehabilitation. 2003;84(4):616–9. 10.1053/apmr.2003.50038 12690603

[47] Bennett L, Kavner D, Lee B, Trainor F. Shear vs pressure as causative factors in skin blood flow occlusion. Archives of physical medicine and rehabilitation. 1979;60(7):309–14. 454129

[48] Loerakker S, Stekelenburg A, Strijkers G, Rijpkema J, Baaijens F, Bader D, et al Temporal effects of mechanical loading on deformation-induced damage in skeletal muscle tissue. Annals of biomedical engineering. 2010;38(8):2577–87. 10.1007/s10439-010-0002-x 20232152 PMC2900588

[49] Coleman S, Gorecki C, Nelson EA, Closs SJ, Defloor T, Halfens R, et al Patient risk factors for pressure ulcer development: systematic review. International journal of nursing studies. 2013;50(7):974–1003. 10.1016/j.ijnurstu.2012.11.019 23375662

[50] Keller PB, Wille J, van Ramshorst B, van der Werken C. Pressure ulcers in intensive care patients: a review of risks and prevention. Intensive care medicine. 2002;28(10):1379–88. 10.1007/s00134-002-1487-z 12373461

[51] Moore Z, Cowman S, Conroy RM. A randomised controlled clinical trial of repositioning, using the 30 tilt, for the prevention of pressure ulcers. Journal of clinical nursing. 2011;20(17‐18):2633–44. 10.1111/j.1365-2702.2011.03736.x 21702861

[52] Theaker C, Kuper M, Soni N. Pressure ulcer prevention in intensive care–a randomised control trial of two pressure‐relieving devices. Anaesthesia. 2005;60(4):395–9. 10.1111/j.1365-2044.2004.04085.x 15766343

[53] Defloor T, De Bacquer D, Grypdonck MH. The effect of various combinations of turning and pressure reducing devices on the incidence of pressure ulcers. International journal of nursing studies. 2005;42(1):37–46. 10.1016/j.ijnurstu.2004.05.013 15582638

[54] Bours G, Laat dE, Halfens R, Lubbers M. Prevalence, risk factors and prevention of pressure ulcers in Dutch intensive care units. Intensive care medicine. 2001;27(10):1599–605. 10.1007/s001340101061 11685300

[55] Defloor T. The effect of position and mattress on interface pressure. Applied nursing research. 2000;13(1):2–11. 10.1016/s0897-1897(00)80013-0 10701278

[56] Heule EJ, Goossens RH, Mugge R, Dietz E, Heule F. Using an Identation Measurement Device to Assess Foam Mattress Quality. Ostomy wound management. 2007;53(11):56. 18057447

[57] Serrano ML, Mendez MG, Cebollero FC, Rodriguez JL. Risk factors for pressure ulcer development in Intensive Care Units: A systematic review. Medicina Intensiva (English Edition). 2017;41(6):339–46. 10.1016/j.medin.2016.09.003 27780589

[58] Tayyib N, Coyer F, Lewis P. Saudi Arabian adult intensive care unit pressure ulcer incidence and risk factors: a prospective cohort study. International wound journal. 2016;13(5):912–9. 10.1111/iwj.12406 25662591 PMC7949994

[59] Cremasco MF, Wenzel F, Zanei SS, Whitaker IY. Pressure ulcers in the intensive care unit: the relationship between nursing workload, illness severity and pressure ulcer risk. Journal of Clinical Nursing. 2013;22(15–16):2183–91. 10.1111/j.1365-2702.2012.04216.x 23216694

[60] Nixon J, Thorpe H, Barrow H, Phillips A, Andrea Nelson E, Mason SA, et al Reliability of pressure ulcer classification and diagnosis. Journal of advanced nursing. 2005;50(6):613–23. 10.1111/j.1365-2648.2005.03439.x 15926966

[61] Gulin FS, Menegueti MG, Auxiliadora-Martins M, de Araujo TR, Bellissimo-Rodrigues F, Nassiff A, et al APACHE II Death Risk and Length of Stay in the ICU Are Associated With Pressure Injury in Critically Ill Patients. Journal of clinical medicine research. 2018;10(12):898 10.14740/jocmr3636 30425762 PMC6225865

[62] Kaitani T, Tokunaga K, Matsui N, Sanada H. Risk factors related to the development of pressure ulcers in the critical care setting. Journal of clinical nursing. 2010;19(3‐4):414–21. 10.1111/j.1365-2702.2009.03047.x 20500281

